# Overexpression of a Gene Encoding Trigonelline Synthase from *Areca catechu* L. Promotes Drought Resilience in Transgenic Arabidopsis

**DOI:** 10.3390/plants11040487

**Published:** 2022-02-11

**Authors:** Yilin Li, Mengying Ding, Chuang Cui, Qiyuan An, Jiao Wu, Guangzhen Zhou, Yinglang Wan, Wenlong Bao

**Affiliations:** 1 Hainan Key Laboratory for Sustainable Utilization of Bioresources, Hainan University, Haikou 570228, China; 15176862705@163.com; 2 College of Tropical Crops, Hainan University, Haikou 570228, China; im_cuichuang@sina.com (C.C.); 18242097267@163.com (Q.A.); wujiao1202@yahoo.com (J.W.); guangzhenvip@163.com (G.Z.); 3 Hainan State Key Laboratory of South China Sea Marine Resources Utilization, College of Marine Science, Hainan University, Haikou 570228, China; ddmying@163.com; 4 College of Horticulture, Hainan University, Haikou 570228, China

**Keywords:** trigonelline synthase, areca seedling, drought resilience, gene expression

## Abstract

*Areca catechu* L. is a commercially important palm tree widely cultured in tropical and subtropical areas. Its growth and production are severely hindered by the increasing threat of drought. In the present study, we investigated the physiological responses of areca seedlings to drought stress. The results showed that prolonged drought-induced yellowing on the overall area of most leaves significantly altered the chlorophyll fluorescence parameters, including maximum chemical efficiency (Fv/Fm), photochemical efficiency of PSII (Y(II)), photochemical chlorophyll fluorescence quenching (qP) and non-photochemical chlorophyll fluorescence quenching (NPQ). On the 10th day of drought treatment, the contents of proline in the areca leaves and roots increased, respectively, by 12.2 times and 8.4 times compared to normal watering. The trigonelline levels in the leaves rose from 695.35 µg/g to 1125.21 µg/g under 10 days of water shortage, while no significant changes were detected in the content of trigonelline in the roots. We determined the gene encoding areca trigonelline synthase (AcTS) by conducting a bioinformatic search of the areca genome database. Sequence analysis revealed that AcTS is highly homologous to the trigonelline synthases in *Coffea arabica* (CaTS 1 and CaTS 2) and all possess a conserved S-adenosyl- L-methionine binding motif. The overexpression of *AcTS* in *Arabidopsis thaliana* demonstrated that AcTS is responsible for the generation of trigonelline in transgenic *Arabidopsis,* which in turn improves the drought resilience of transgenic *Arabidopsis.* This finding enriches our understanding of the molecular regulatory mechanism of the response of areca to water shortage and provides a foundation for improving the drought tolerance of areca seedlings.

## 1. Introduction

Drought presents an increasing threat to plant survival and distribution due to global climate change. Drought stress antagonistically regulates plant growth and development via environmental stimuli including elevated temperature, high salinity and limited water availability [1,2,3,4]. Plants are sessile and have evolved an advanced drought-responsive mechanism to deal with drought stress. The abscisic acid (ABA) signaling pathway in plants is the core mechanism for modulating leaf transpiration and water content by regulating stomatal closure under drought stress [5,6,7]. In concert with stomatal closure, the impermeable cuticle covering plant leaves acts as a crucial transpiration barrier that reduces water loss from plant leaves, thereby helping plants to survive drought conditions [8,9,10]. At the phenotypic level, plants can enhance their water uptake by regulating the root length, root width and root density to preserve the physiological water balance of the cells under water-deficit circumstances [11,12]. Osmotic adjustment is a highly efficient strategy adopted by plants to alleviate drought-induced osmotic damage to cells at the cellular level, playing a pivotal role in plant adaptation to drought stress [13,14]. During drought spells, plants actively produce diverse osmoprotectants (including soluble sugars, proline, glycine betaine and trigonelline) within cells to sustain normal turgor pressure and improve the capacity for cell water absorption or water retention to adapt to water shortage [15,16,17,18]. The self-protective strategies adopted by plants against drought stress are multidimensional, and deciphering these complicated regulation networks is necessary for furthering our understanding of drought resilience in plants.

N-methyl nicotinate, a pyridine derivative with diverse biological functions, is also known as trigonelline due to its first isolation from a legume crop called *Trigonella foenum-graecum* L. [17,19]. Comparable to natural phytohormones, trigonelline can significantly affect cell proliferation by arresting cells in the interphase of the cell cycle [20,21]. Chen et al. found that the overaccumulation of trigonelline in rice was highly correlated with wider rice grains, and this metabolite–phenotype association phenomenon was caused by trigonelline arresting cells in the G2/M phase and extending the whole cell cycle [22]. Mazzuca et al. [23] revealed that trigonelline perturbs DNA replication by inhibiting the ligation of replicons in the S-phase of the cell cycle, thus impairing the root elongation of lettuce, which implies that trigonelline also acts as a cell cycle regulator. DNA hypomethylation induced by trigonelline, which is generally implicated in DNA replication-associated cell cycle arrest, may play a crucial role in the regulatory effects of trigonelline on the cell cycle [24,25]. However, the function of trigonelline-mediated hypomethylation on the cell cycle has not been further detailed. Additionally, trigonelline works as a signal transmitter in diverse processes such as circadian rhythm, nodulation and oxidative stress [20]. It is noteworthy that trigonelline—as a ubiquitous osmolyte—helps plants to challenge abiotic stress by sustaining turgor pressure, protecting the integrity of the plasma membrane and stabilizing the structure and function of proteins [17,26,27]. However, a fine-tuned regulatory mechanism of trigonelline in response to abiotic stress lacks definitive evidence at the molecular level.

Trigonelline is derived from nicotinic acid (NA), which is produced by the pyridine nucleotide cycle and is considered to be a reservoir of NA for detoxifying the cytotoxicity caused by the overaccumulation of NA. In planta, trigonelline synthase (N-methyltransferase) uses S-adenosyl-L-methionine (SAM) as a methyl group donor and NA as an acceptor to catalyze the terminal step of trigonelline biosynthesis [15,28]. Trigonelline synthase was first found in the crude extract of *Pisum sativum* and has been purified from *Glycine max* [29]. Mizuno et al. first reported that two caffeine synthase (CCS)-homologous genes in *Coffea arabica* encode coffee trigonelline synthases (CTgS1 and CTgS2) [19]. Li et al. demonstrated that protein NA-N-methyltransferase (AtNANMT1) encoded by *At3g53140* is principally responsible for trigonelline production in *Arabidopsis thaliana,* and caffeic acid O-methyltransferase (COMT) encoded by *At5g54160* has weak NANMT activity for catalyzing the methylation of NA [28]. Interestingly, *CTgS1* and *CTgS2* belong to the *SABATH* gene family and share 82% identity with *CCS*, while no *AtNANMT1* homologs have been found in the coffee genome [28]. These findings suggest that different types of methyltransferases may be involved in trigonelline biosynthesis.

*Areca catechu* L. is a commercially important plant species grown in tropical and subtropical areas around the world. It originated in the Malaysian peninsula and is widely cultivated throughout the south and southeast of Asia. The fruits of areca, also known as betel nuts, have been used as a food and medicine for thousands of years and are estimated to be consumed by about 600 million individuals at present [30,31,32]. Areca thrives best in a humid environment, and drought conditions induce severe retardation of growth and production. However, the drought response of areca is not well understood. We obtained the gene encoding areca trigonelline synthase (AcTS) by conducting a bioinformatic search of the areca genome database (AC10G024220; id: JAHSVC000000000; BioSample: SAMN19591864). The full-length CDS sequences of *AcTS* were cloned from areca and overexpressed in *Arabidopsis thaliana*. The results demonstrated that the overexpression of *AcTS* improves the drought tolerance of transgenic *Arabidopsis* by regulating the production of trigonelline. This discovery enriches our understanding of the molecular regulatory mechanism of the response of areca to water shortage, providing a foundation for improving the drought resilience of areca.

## 2. Results

### 2.1. Drought Stress Response of A. catechu Seedlings

**Figure 1:**
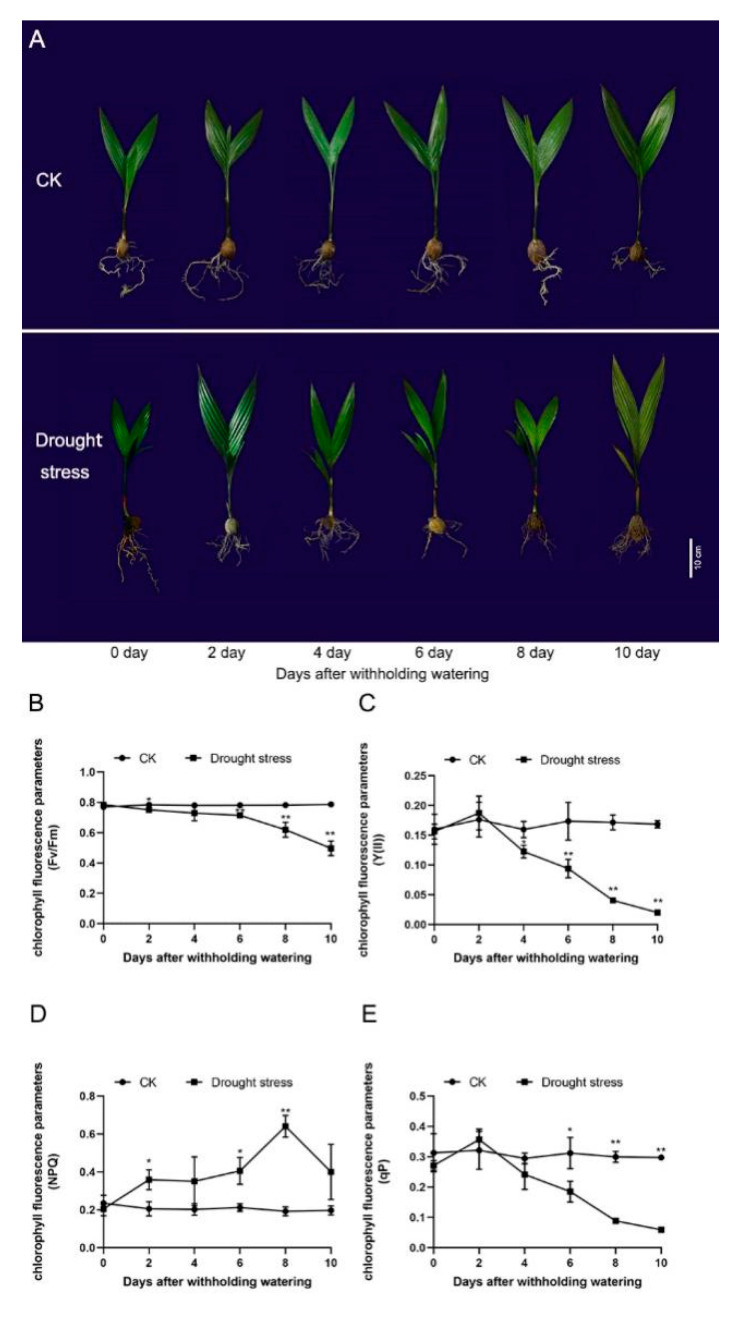
Growth of the areca seedlings under treatment (*n* = 3). (**A**) Phenotype of the areca seedlings. Scale bar = 10 cm. (**B**) The value of Fv/Fm. (**C**) The value of Y(II). (**D**) The value of qP. (**E**) The value of NPQ. Data are expressed as the means ± standard errors of the means. * *p* value < 0.05; ** *p* < 0.01.

Water shortage had a significant effect on the morphology of the *A*. *catechu* seedlings. As water content decreased in the leaf and root of areca seedlings (Supplementary Figure S3A,B), on the 4th day-after-treatment (DAT), yellowing on the outer edge of the leaves occurred and the overall area of most leaves had turned yellow by the 10th DAT (Figure 1A). The longer the treatment, the greater the decrease in the chlorophyll fluorescence parameters, including maximum chemical efficiency (Fv/Fm), photochemical efficiency of PSII (Y(II)) and photochemical chlorophyll fluorescence quenching (qP). Fv/Fm significantly decreased to 0.75 on the 2nd DAT, while Y(II) and qP remarkably declined to 0.12 and 0.24, respectively, on the 4th DAT. On the 10th DAT, Fv/Fm, Y(II) and qP decreased by 36.8%, 87.9% and 80.1%, respectively (Figure 1D–F). As the duration of water deficit increased, the non-photochemical chlorophyll fluorescence quenching (NPQ) gradually ascended and peaked on the 8th DAT, decreasing dramatically thereafter (Figure 1E).

### 2.2. Changes in Trigonelline and Proline in A. catechu Seedlings under Drought Stress

**Figure 2:**
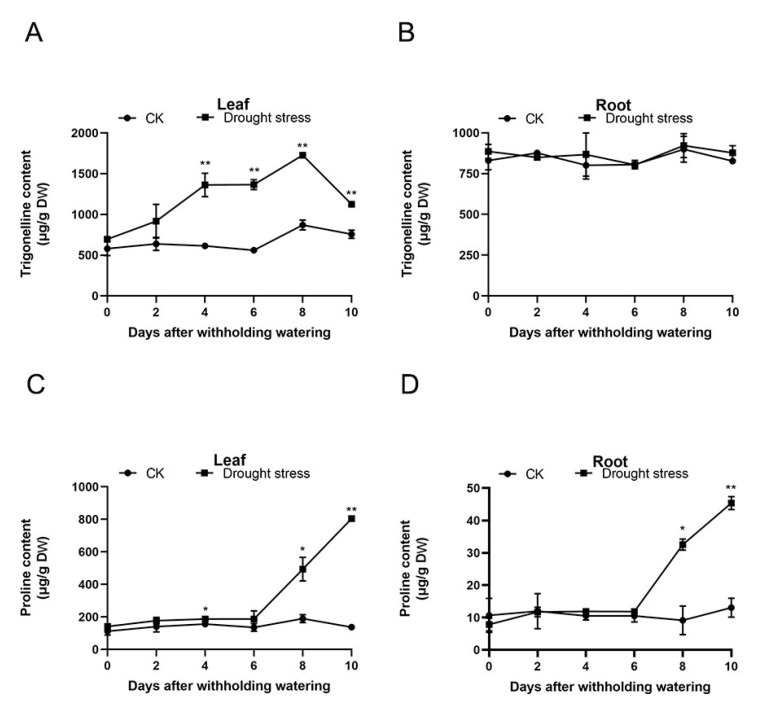
The content of trigonelline and proline in the areca leaves and roots (*n* = 3). (**A**) The content of trigonelline in the areca leaves. (**B**) The content of trigonelline in the areca roots. (**C**) The content of proline in the areca leaves. (**D**) The content of proline in the areca roots. DW: dry weight. Data are expressed as the means ± standard errors of the means. * *p* value < 0.05; ** *p* < 0.01.

With prolonged treatment, the content of trigonelline in the areca leaves dramatically increased from the 4th DAT and peaked on the 8th DAT. On the 10th DAT, the trigonelline levels decreased by 1.5 times compared with those on the 8th DAT, though the values were still significantly higher than that on day zero (Figure 2A). As shown in Figure 2B, 10 days of water shortage had no significant effect on the content of trigonelline in the areca roots. The content of proline in the leaves markedly ascended on the 4th DAT. On the 10th DAT, the proline levels in the leaves reached 804.98 µg/g DW, which was 5.88-fold higher than that in the controls (Figure 2C). The content of proline in the areca roots sharply changed on the 8th DAT and continuously increased on the 10th DAT. On the 10th DAT, the proline levels in the areca roots reached 45.35 µg/g DW, which was 3.47-fold higher than that in the controls (Figure 2D).

### 2.3. Defining the Trigonelline Synthesis Gene from the A. catechu Genome

**Figure 3:**
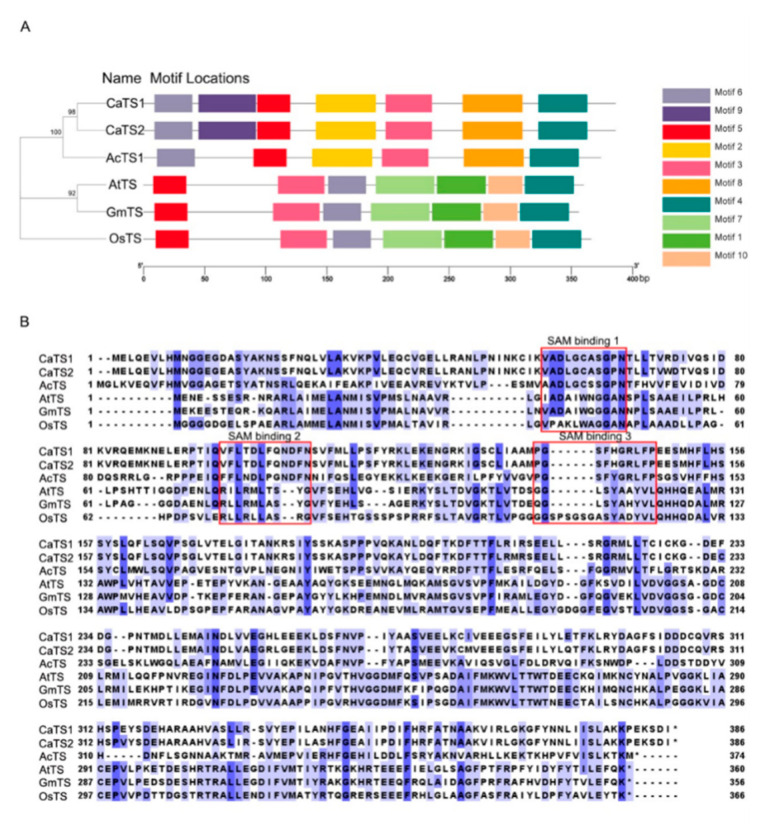
Sequence analysis of trigonelline synthases from different plant species. (**A**) Phylogenetic tree and conserved motifs of AcTS, CaTS1, CaTS2, AtTS, GmTS and OsTS. Different motifs are represented by different boxes. The location of each motif can be estimated using the scale at the bottom. (**B**) Alignment of amino acid sequences of AcTS, CaTS1, CaTS2, AtTS, GmTS and OsTS. Three proposed SAM regions are shown in the open boxes. AcTS: *Areca catechu* trigonelline synthase; AtTS: *Arabidopsis thaliana* trigonelline synthase; OsTS: *Oryza sativa* trigonelline synthase; CaTS: *Coffea arabica* trigonelline synthase; GmTS: *Glycine max* trigonelline synthase.

**Figure 4:**
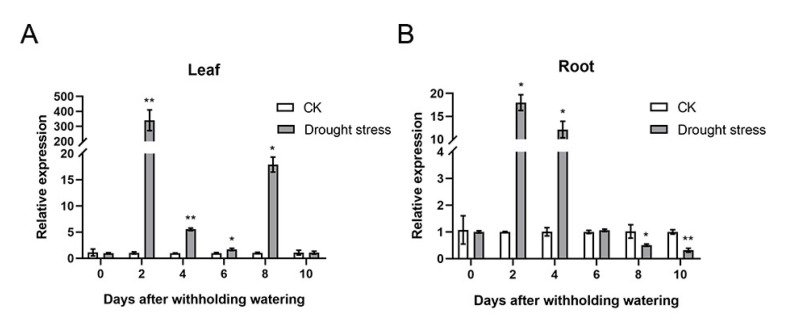
The transcript abundance of *AcTS* in areca leaves and roots under treatment (*n* = 3). (**A**) Transcript abundance of *AcTS* in acrea leaves under treatment. (**B**) Transcript abundance of *AcTS* in acrea roots under treatment. Data are expressed as the means ± standard errors of the means. * *p*-value < 0.05; ** *p* < 0.01.

To investigate the evolutionary relationships of trigonelline synthase in different plant species, we constructed a phylogenetic tree based on the full-length amino acid sequences of *A. catechu* trigonelline synthase (AcTS; id: JAHSVC000000000; BioSample: SAMN19591864), *A. thaliana* trigonelline synthase (AtTS; NM_115174, BioProject: PRJNA116), *Oryza sativa* trigonelline synthase (OsTS; Q6K9 × 3, BioProject: PRJNA122), *Coffea arabica* trigonelline synthase (CaTS; AB054842 and AB054843; BioProject: PRJNA497895) and *Glycine max* trigonelline synthase (GmTS; NM_001360031, BioProject: PRJNA19861). The results showed that those trigonelline synthases from different plant species could be categorized into three subgroups, in which AcTS and CaTS (CaTS1 and CaTS2) were clustered into the same subgroup and AtTS and GmTS gathered together, while OsTS was grouped into a separate clade (Figure 3A). The MEME analysis revealed that motif 3, motif 4, motif 5 and motif 6 were present in all trigonelline synthases from different plant species, while motif 9 was specifically present in the N-terminal region of CaTS (CaTS1 and CaTS2) compared with AcTS in the same subgroup. In addition, the trigonelline synthases in different subgroups had different specific motifs, which was in line with their phylogenetic clustering (Figure 3A). The *TS* gene from different plant species consisted of different lengths of CDSs (*AcTS*: 1122 bp, *CaTS1*: 1158 bp, *CaTS2*: 1158 bp, *AtTS*: 1080 bp, *GmTS*: 1068 bp and *OsTS*: 1098 bp), which encoded different lengths of amino acid residues (AcTS: 373 aa, CaTS1: 385 aa, CaTS2: 385 aa, AtTS: 359 aa, GmTS: 355 aa and OsTS: 365 aa) (Figure 3B and Supplementary seq 1 to 12). The alignment of the amino acid sequences of TS revealed that AcTS, CaTS1 and CaTS2 all contained the proposed SAM binding motifs (SAM binding 1, SAM binding 2 and SAM binding 3) (Figure 3B). To test the expression patterns of *AcTS* under drought, the transcript abundance of *AcTS* in the areca leaves and roots was determined via quantitative real-time PCR (qRT-PCR). With prolonged treatment, the expression level of *AcTS* remarkably increased in both the leaves and roots on the 2nd DAT, with expression levels that were ~80-fold and ~6-fold higher than that in the controls, respectively (Figure 4A,B). On the 4th and 6th DAT, the expression level of *AcTS* in the leaves were lower than that on the 2nd DAT, while the level was still significantly higher in the treatment group than that in the controls. On the 8th DAT, the expression level of *AcTS* was elevated in comparison with that on the 4th and 6th DAT, while the level was still lower than that on the 2nd DAT. On the 10th DAT, the expression level of *AcTS* sharply dropped to the level of no significant difference between the control and treatment groups (Figure 4A). In the roots, the expression pattern of *AcTS* on the 4th and 6th DAT were similar to that in the leaves. From the 8th DAT, *AcTS* was significantly downregulated in the treatment group (Figure 4B).

### 2.4. Overexpression of AcTS Increased the Drought Tolerance of Arabidopsis

**Figure 5:**
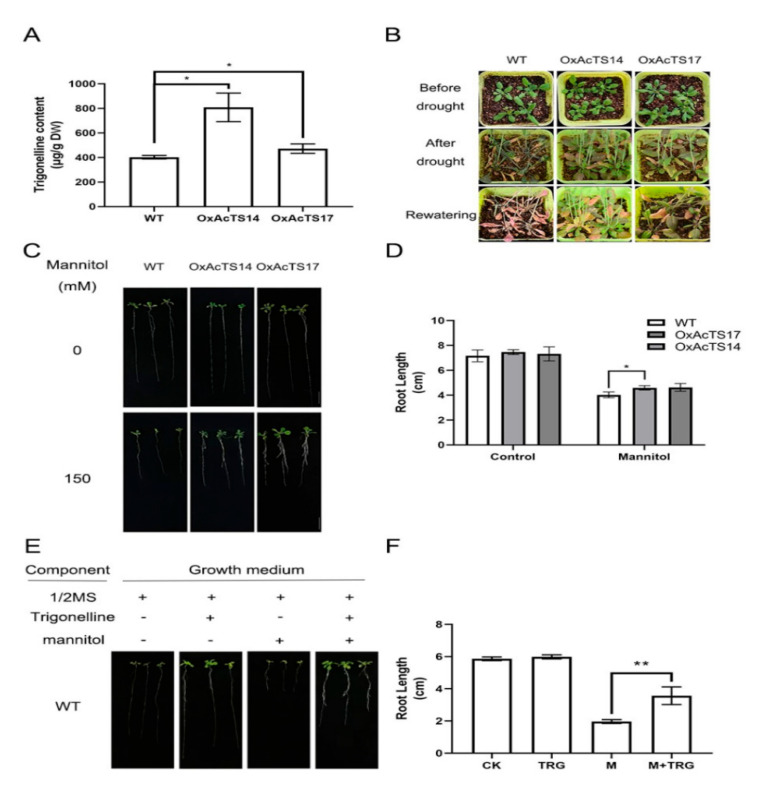
The potential role of AcTS in response to drought. (**A**) The content of trigonelline in Col-0 and transgenic *Arabidopsis* overexpressing *AcTS* (OxAcTS14 and OxAcTS14). (**B**) The phenotype of Col-0 and OxAcTS under drought and rehydration treatments. (**C**) The phenotype of Col-0 and OxAcTS on half-strength MS medium and half-strength MS medium supplemented with mannitol. (**D**) The root length of Col-0 and OxAcTS on half-strength MS medium and half-strength MS medium supplemented with mannitol. (**E**) The phenotype of Col-0 on half-strength MS medium, half-strength MS medium supplemented with trigonelline, half-strength MS medium supplemented with mannitol and half-strength MS medium supplemented with trigonelline and mannitol. A minus (**−**) sign indicates that the growth medium lacked the corresponding component, and a plus (+) sign indicates that the growth medium was supplemented with the corresponding ingredients. (**F**) The root length of Col-0 on half-strength MS medium, half-strength MS medium supplemented with trigonelline, half-strength MS medium supplemented with mannitol and half-strength MS medium supplemented with trigonelline and mannitol. Data are expressed as the means ± standard errors of the means. * *p* value < 0.05; ** *p* < 0.01.

To evaluate the role of AcTS in the drought response, transgenic *Arabidopsis* plants overexpressing *AcTS* (OxAcTS) were generated using the *Agrobacterium* infection method. As shown in Figure 5A, the contents of trigonelline in *OxAcTS14* and *OxAcTS17* were ~2-fold and ~1.2-fold higher than that of the wild-type Col-0, respectively. After 16 days of drought treatment, Col-0 showed either a severe yellowing phenotype or necrosis and failed to recover after 4 days of rehydration (Figure 5B). We found that the T3 generation of OxAcTS (OxAcTS14 and OxAcTS17) showed slight wilting after 16 days of drought, and the plants recovered quickly after 4 days of rehydration (Figure 5B). On half-strength Murashige and Skoog (MS) medium, there was no great difference in root length between Col-0 and OxAcTS (Figure 5C,D). On half-strength MS medium supplemented with mannitol, Col-0 and OxAcTS had comparatively shorter roots than those on half-strength MS medium, whereas the root length of OxAcTS was significantly longer than that of Col-0 (Figure 5C,D). On half-strength MS medium supplemented with trigonelline, Col-0 had larger leaves and longer roots than those on half-strength MS medium. On half-strength MS medium supplemented with mannitol and trigonelline, Col-0 had stronger and longer roots in comparison with those on half-strength MS medium supplemented with mannitol (Figure 5E,F).

## 3. Discussion

As tropical regions experience different annual dry and wet seasons, areca trees have developed a complex system to adapt to drought and flood stresses. The two-season rhythm in tropical regions are quite different from the four-season rhythm in subtropical and temperate zones. Accordingly, studies on the drought tolerance mechanisms of tropical plants can broaden our understanding of the evolution of drought tolerance systems in plant realms. *Areca catechu* is one of the most widely distributed palm trees in tropical and subtropical regions. Here, we analyzed how drought affected the photosynthetic activities of the leaves of areca seedlings.

The chlorophyll fluorescence parameters including Fv/Fm, Y(II), qP and NPQ reflect the photochemical properties and functionalities of plants and are considered as crucial indicators for evaluating the effect of the environmental conditions on photosynthetic activities [33,34,35,36,37]. Fv/Fm represents the maximal photochemical efficiency of PS II in dark-adapted samples and reflects the photosynthetic performance of plants. Y(II) refers to the actual photochemical quantum yield of PS II in illuminated samples, which is related to the non-cyclic electron transport used for photochemical reactions. Furthermore, qP reflects the degree of openness of the PS II reaction centers and oxidized state of the primary electron acceptor (plastoquinone acceptor, QA). Y(II) and qP in the drought treatment increased with no significant difference compared to the control at 2 days after withholding watering, indicating that areca seedlings were able to regulate the photochemical conversion efficiency to maintain normal photosynthesis at the early stage of water deficit. However, Y(II) and qP in the drought treatment rapidly decreased from the 4th DAT, indicating that prolonged water deficit caused significant damage to the photosynthesis capacity of areca seedlings. Reads of Fv/Fm gradually decreased during the drought treatment, implying that the photosynthetic activities were inhibited by drought. NPQ is a photoprotective regulatory mechanism used by plants against photodamage and acts by thermally dissipating the excess light energy that cannot be utilized by the photosynthetic apparatus. The peak value of NPQ occurred eight days after drought treatment and decreased dramatically thereafter. The increase in *NPQ* in water-starved areca leaves was inversely related to the trend of Fv/Fm, Y(II) and qP, suggesting that drought stress caused the impairment of the photosynthetic apparatus and weakened the capability of the photosynthetic apparatus to utilize the excitation energy. The dramatic decrease in photosynthetic performance suggested that continuous drought may have disordered the heat-releasing mechanisms in the chloroplasts.

Areca trees have been cultivated for areca nuts, as these produce a stimulant that is derived from the alkaloids synthesized in almost all types of cells in the plant body [38]. The *Areca* genus may contain the highest amounts of alkaloids among all palm trees [31,39,40]. Though the psychologic and pharmaceutic activities of arecoline and its variant alkaloids have been well studied, little is known about the physiological activities of alkaloids in areca trees. Cho et al. [26] reported that the trigonelline level in different soybean genotypes was markedly affected by the degree of soil moisture experienced by the seedlings. Our results support this finding and contribute to our understanding of the physiological roles of alkaloids in palm trees.

Among all drought tolerance responses, the accumulation of osmolytes to alleviate the adverse effects of drought is a common approach [41,42,43]. Proline is a widely used biochemical marker in plant drought-tolerant responses and is an antioxidant osmolyte that adjusts osmotic pressure and stabilizes protein structures in plants. Drought stress will increase the proline content in plant cells [16,44]. Our study corroborates this, as the contents of proline in the leaves dramatically changed on the 4th DAT and increased with the duration of water shortage (Figure 2C). Trigonelline content in leaves increased significantly at 4 days of drought, peaked on the 8th DAT and then decreased on the 10th DAT (Figure 2A), suggesting that trigonelline was induced by drought and might in turn regulate drought resistance of plants.

The conversion of nicotinic acid to trigonelline is catalyzed by an N-methyltransferase, namely trigonelline synthase (TS) [15,28]. Trigonelline exists in many plants, including *Coffea Arabica*, *Trifolium incamatum*, *Medicago sativa and Glycine max*. Here, we obtained a series of genes annotated to N-methyltransferase from the whole genomic database (AC10G024220; id JAHSVC000000000, BioSample: SAMN19591864) and transformed them into the model plant *A. thaliana.* The results indicated that only one of them increased the endogenous trigonelline content, confirming that this sequence is a trigonelline synthase gene, which we named *AcTS.* The expression level of *AcTS* increased dramatically in both the leaves and roots of the areca seedlings after 2 days of drought treatment. However, the expression level in the leaves increased by about 80 times compared with that under standard watering treatment, while the expression level in the roots increased by only about 6 times. This may explain why the trigonelline contents increased mostly in the leaves. The motif analysis identified AcTS as a closely related homologue to coffee trigonelline synthases (CaTS1 and CaTS2), which are N-methyltransferases catalyzing the terminal step of trigonelline biosynthesis [15,28]. Meanwhile, AtTS, GmTS and OsTS shared similar structural information with another clade, the O-methyltransferases, which have weak NANMT activity for catalyzing the methylation of NA. Since areca tree, maize and rice are monocotyledons, while coffee and *Arabidopsis* are dicotyledons, the TS in different plant groups might have evolved independently.

The transgenic *Arabidopsis* lines overexpressing *AcTS* provided us another opportunity to evaluate the role of trigonelline in drought tolerance. Mannitol was added to the culture medium to mimic drought tolerance. We found that both the additional trigonelline and overexpressed *AcTS* increased the drought tolerance of *Arabidopsis.* Since AtTS has only weak NANMT activity, the transgenic *Arabidopsis* lines showed higher trigonelline levels than Col-0 and rapidly recovered from the severe drought treatment after rehydration. Moreover, the *Arabidopsis* WT seedlings with extra exogenous trigonelline and the overexpression line of *Arabidopsis* with endogenous *AcTS* expression exhibited a similar phenomenon; for instance, longer roots and better-developed lateral roots. This again confirmed that the overexpression of *AcTS* in *Arabidopsis* increased the drought tolerance of *Arabidopsis* by producing trigonelline in the plants.

In conclusion, we analyzed the physiological parameters of areca seedlings including chlorophyll fluorescence parameters together with proline and trigonelline content to drought stress. We found that trigonelline, a new alkaloid in *Areca catechu* observed in our previous work [45], greatly accumulated in areca seedlings under drought. To demonstrate the possible role of trigonelline in drought tolerance, the *AcTS* gene was cloned and overexpressed in *Arabidopsis thaliana* where it was found to improve drought resilience by regulating the production of trigonelline. Our study lays a foundation for further exploring the molecular regulatory mechanism of *AcTS* gene in plant drought resistance.

## 4. Materials and Methods

### 4.1. Plant Materials and Treatment

We acquired areca seedlings from the Coconut Research Institute of the Chinese Academy of Tropical Agricultural Sciences, Wenchang, Hainan province, China. The uniformly grown 2-leaf areca seedlings were transferred to plastic pots (size: 12 cm × 12 cm) containing sterilized perlite, which were placed in a plant growth incubator (light cycle: 16 h of light and 8 h of darkness; temperature: 28 °C, relative humidity: 50%) at Hainan University, Haikou, Hainan province, China. The seedlings were irrigated with half-strength Hoagland’s nutrient solution (pH = 6.0) every week for 2 weeks. The seedlings were then classified into two groups: the control group, wherein the seedlings were watered normally; and the treatment group, wherein the seedlings underwent 10 consecutive days of no water to simulate drought (Supplementary Figure S1). The roots and the first leaf from the top of each seedling (Supplementary Figure S2) were collected at 0, 2, 4, 6, 8 and 10 days after treatment, immediately frozen in liquid nitrogen and stored at −80 °C until use. All experiments were replicated at least three times.

Different growth media were used to cultivate *A. thaliana* plants, including half-strength MS medium, half-strength MS medium supplemented with 150 mM mannitol, half-strength MS medium supplemented with 1 mg/L trigonelline and half-strength MS medium supplemented with 150 mM mannitol and 1 mg/L trigonelline. The primary root length of the plants was measured after the 10-day old *Arabidopsis* seedlings cultivated on half-strength MS medium had been transferred to different growth media to grow for 7 days. For the drought resistance test, the 10-day-old *Arabidopsis* seedlings cultivated on half-strength MS medium were transferred to plastic pots containing a mixture of peat soil and vermiculite at a 3:1 ratio by volume and then treated with drought for 14 days, followed by recovery irrigation for 4 days.

### 4.2. Determination of Chlorophyll Fluorescence Parameters

When the areca seedlings were in the growth stage of two true leaves, chlorophyll fluorescence parameters of the first leaf from the top of each seedling were measured simultaneously using a Dual-PAM-100 Dual channel modulated chlorophyll fluorescence instrument (WALZ, Germany), including maximum chemical efficiency (Fv/Fm, photochemical efficiency of PSII (Y(II)), photochemical fluorescence quenching (qP) and non-photochemical quenching (NPQ). Prior to measurements, all plants were placed under dark conditions for more than 30 min. Thereafter, the chlorophyll fluorescence induction curve was determined in the ‘Fluo + P700’ mode. Three points of each leaf were chosen for measurement, and each treatment was repeated at least three times.

### 4.3. Determination of Trigonelline Content

Fifty percent methanol solution was added to the 50 mg plant sample and vortexed to dissolve any residue. The mixtures were subjected to ultrasonic treatment for 30 min and then placed in a refrigerator at −20 °C for 3–5 h. After cooling, the mixtures were thoroughly vortex-mixed and centrifuged at 10,000 rpm for 10 min to obtain a supernatant solution. This supernatant solution was filtrated by microporous membrane filtration (0.22 µm) into a new vial. Concentrations of 5, 10, 12.5, 25 and 50 µg/mL trigonelline standard solutions were prepared for drawing the standard curve. A high-performance chromatography (HPLC) system (e2695, Waters, MA, USA) with a diode array detector (2998, Waters, MA, USA) was utilized for the trigonelline analysis. Samples (10 µL) were separated using a cation-exchange column (300 SCX, 250 mm × 4.6 mm × 5 µm, Agilent, CA, USA) at 35 °C. The mobile phase contained a mixture of 0.2% phosphoric acid (adjusted to pH 3.7 with NH4OH) and acetonitrile in a 70:30 ratio by volume. The flow rate was 1 mL/min and the eluent was monitored at 215 nm.

### 4.4. Determination of Proline Content

Frozen leaves (0.1 g) were homogenized in 3% sulphosalicylic acid and transferred to a tube at a constant volume of 5 mL. The homogenate was heated at 100 °C for 5 min and then filtered. Filtrate (0.5 mL) was placed into a new micro-tube to which 1.5 mL of water, 2 mL of ninhydrin and 2 mL of glacial acetic acid were added, followed by treatment in boiling water bath for 30 min. After cooling at room temperature, the mixtures were combined with 5 mL toluene to extract chromophores in the dark for 2–3 h. The absorbance was read at a wavelength of 520 nm using a microplate reader (TECAN, Switzerland).

### 4.5. Sequence Analysis of the Gene Encoding Trigonelline Synthase

The MEME (http://meme.nbcr.net/meme/intro.html (accessed on 12 July 2021)) and TBtools (Gene Structure View (Advanced)) platforms were used to analyze and visualize the conserved motifs in the amino acid sequences of trigonelline synthase (TS). All settings were at default values. The maximum-likelihood method in MEGA X software was used to construct the phylogenetic tree with 10,000 bootstrap replications. The source sequences were as follows: *A.* *catechu* TS (accession number: AC10G024220; id: JAHSVC000000000; BioSample: SAMN19591864), *A.* *thaliana* TS (Accession number: NM_115174; BioProject: PRJNA116), *O.* *sativa* TS (Accession number: Q6K9 × 3; BioProject: PRJNA122), *C.* *arabica* TS (Accession number: AB054842 and AB054843; BioProject: PRJNA497895) and *G.* *max* TS (Accession number: NM_001360031; BioProject: PRJNA19861). The online software Clustal Omega (https://www.ebi.ac.uk/Tools/msa/clustalo/ (accessed on 12 July 2021)) was used to carry out the multiple sequence alignment (default parameters), and Jalview software was used to edit the background color, fonts and format.

### 4.6. Extraction of Total RNA

Total RNA of the plant samples was extracted using a plant RNA extraction kit (TIANGEN, Beijing, China), as described by the manufacturer. The RNA concentration and quality were detected by a NanoDrop 2000 (KAIAO, Beijing, China) and agarose gel electrophoresis.

### 4.7. Cloning of the Full-Length AcTS CDS

The high-quality total RNA was then used for cDNA synthesis using the RNAprep Pure Plant Kit (TIANGEN, Beijing, China). The full-length CDS of the trigonelline synthase gene from *A.* *catechu* was cloned using a FastKing RT Kit (TIANGEN, Beijing, China) under the following PCR conditions: pre-denaturation of 5 min at 94 °C, amplification of 35 cycles at 94 °C for 30 s, 60 °C for 30 s and 72 °C for 1 min and final extension of 10 min at 72 °C. The primer sequences were designed using SnapGene software and are listed in Supplementary Table S1.

### 4.8. Analysis of Quantitative Real Time PCR (qRT-PCR)

The qRT-PCR assay was carried out using 2 × ChamQ Universal SYBR qPCR Master Mix Kit (Vazyme, Nanjing, China) and a BIO-RAD Real-time fluorescence PCR instrument (BIO-RAD, CA, USA). The qRT-PCR reaction system contained 25 ng cDNA, 1 µM of each primer, 10 µL 2 × ChamQ Universal SYBR qPCR Master Mix (Vazyme, Nanjing, China) and 7.8 µL RNAase-free water. The qRT-PCR programming was as follows: denaturation at 95 °C for 30 s followed by 40 amplification cycles (95 °C for 10 s and 60 °C for 30 s). *Actin* was used as an internal housekeeping gene. All primer sequences were designed using Premier 5 software and are listed in Supplementary Table S2.

### 4.9. Generation of Transgenic Arabidopsis Overexpressing AcTS

The full-length AcTS CDS was inserted into the KpnI and BamHI sites of the pCAMBIA1300 vector to obtain the overexpression construct according to standard molecular biology methods. Approximately 1 µg of the overexpression construct and pCAMBIA1300 empty vector were transformed into *Agrobacterium tumefaciens* (strain GV3101). The correct *Agrobacterium* transformants were then transformed into the wild-type *Arabidopsis* (Col-0) to generate transgenic *Arabidopsis* overexpressing AcTS (OxAcTS) via the floral-dip method as described in a previously published article [46]. Two homozygous T3 transgenic lines (OxAcTS14 and OxAcTS17) with high overexpression of AcTS were selected for further study.

### 4.10. Statistical Analysis

The Student’s *t* test and one-way ANOVA analyses were carried out using SPSS Statistics 26 (IBM Corp., Armonk, NY, USA). The values are shown as the mean ± standard deviation. Origin 2018 and GraphPad Prism 8.0.2 software were used to draw the graphs.

## Data Availability

The datasets presented in this study can be found in online repositories. The names of the repository/repositories and accession number(s) can be found in the article/Supplementary Material.

## Supplementary Materials

The following supporting information can be downloaded at: https://www.mdpi.com/article/10.3390/plants11040487/s1, Figure S1: The flow chart of this study; Figure S2: Schematic diagram of areca seedling; Figure S3: The water content in the leaf and root of areca seedling. Table S1: The primers used for PCR; Table S2: The primers used for qRT-PCR; Letter S1: The full length CDS sequences and amino acid sequences of TS of different species.

## References

[1] Zhu J.K. (2002). Salt and drought stress signal transduction in plants. Annu. Rev. Plant Biol..

[2] Ahuja I., de Vos R.C.H., Bones A.M., Hall R.D. (2010). Plant molecular stress responses face climate change. Trends Plant Sci..

[3] Gupta A., Rico-Medina A., Caño-Delgado A.I. (2020). The physiology of plant responses to drought. Science.

[4] Chávez-Arias C.C., Ligarreto-Moreno G.A., Ramírez-Godoy A., Restrepo-Díaz H. (2021). Maize responses challenged by drought, elevated daytime temperature and arthropod herbivory stresses: A physiological, biochemical and molecular view. Front. Plant Sci..

[5] Schroeder J.I., Kwak J.M., Allen G.J. (2001). Guard cell abscisic acid signalling and engineering drought hardiness in plants. Nature.

[6] Djebbar R., Rzigui T., Pétriacq P., Mauve C., Priault P., Fresneau C., De Paepe M., Florez-Sarasa I., Benhassaine-Kesri G., Streb P. (2012). Respiratory complex I deficiency induces drought tolerance by impacting leaf stomatal and hydraulic conductances. Planta.

[7] Mega R., Abe F., Kim J.S., Tsuboi Y., Tanaka K., Kobayashi H., Sakata Y., Hanada K., Tsujimoto H., Kikuchi J. (2019). Tuning water-use efficiency and drought tolerance in wheat using abscisic acid receptors. Nat. Plants.

[8] Yeats T.H., Rose J.K.C. (2013). The formation and function of plant cuticles. Physiol. Plant..

[9] Xue D.W., Zhang X.Q., Lu X.L., Chen G., Chen Z.H. (2017). Molecular and Evolutionary Mechanisms of Cuticular Wax for Plant Drought Tolerance. Front. Plant Sci..

[10] Patwari P., Salewski V., Gutbrod K., Kreszies T., Dresen-Scholz B., Peisker H., Steiner U., Meyer A.J., Schreiber L., Dormann P. (2019). Surface wax esters contribute to drought tolerance in Arabidopsis. Plant J..

[11] Bhaskarla V., Zinta G., Ford R., Jain M., Varshney R.K., Mantri N. (2020). Comparative root transcriptomics provide insights into drought adaptation strategies in chickpea (*Cicer arietinum* L.). Int. J. Mol. Sci..

[12] Boguszewska-Mańkowska D., Zarzyńska K., Nosalewicz A. (2020). Drought differentially affects root system size and architecture of potato cultivars with differing drought tolerance. Am. J. Potato Res..

[13] Blum A. (2017). Osmotic adjustment is a prime drought stress adaptive engine in support of plant production. Plant Cell Environ..

[14] Turner N.C. (2018). Turgor maintenance by osmotic adjustment: 40 years of progress. J. Exp. Bot..

[15] Ashihara H. (2008). Trigonelline (N-methylnicotinic acid) biosynthesis and its biological role in plants. Nat. Prod. Commun..

[16] Loukehaich R., Wang T.T., Ouyang B., Ziaf K., Li H.X., Zhang J.H., Lu Y.E., Ye Z.B. (2012). SpUSP, an annexin-interacting universal stress protein, enhances drought tolerance in tomato. J. Exp. Bot..

[17] Perchat N., Saaidi P.L., Darii E., Pellé C., Petit J.L., Besnard-Gonnet M., de Berardinis V., Dupont M., Gimbernat A., Salanoubat M. (2018). Elucidation of the trigonelline degradation pathway reveals previously undescribed enzymes and metabolites. Proc. Nat. Acad. Sci. USA.

[18] Ozturk M., Unal B.T., García-Caparrós P., Khursheed A., Gul A., Hasanuzzaman M. (2021). Osmoregulation and its actions during the drought stress in plants. Physiol. Plant..

[19] Mizuno K., Matsuzaki M., Kanazawa S., Tokiwano T., Yoshizawa Y., Kato M. (2014). Conversion of nicotinic acid to trigonelline is catalyzed by N-methyltransferase belonged to motif B′ methyltransferase family in Coffea arabica. Biochem. Biophys. Res. Commun..

[20] Minorsky P.V. (2003). The Hot and the Classic. Plant Physiol..

[21] Sasamoto H., Ashihara H. (2014). Effect of nicotinic acid, nicotinamide and trigonelline on the proliferation of lettuce cells derived from protoplasts. Phytochem. Lett..

[22] Chen W., Wang W.S., Peng M., Gong L., Gao Y.Q., Wan J., Wang S.C., Shi L., Zhou B., Li Z.M. (2016). Comparative and parallel genome-wide association studies for metabolic and agronomic traits in cereals. Nat. Commun..

[23] Mazzuca S., Bitonti M.B., Innocenti A.M., Francis D. (2000). Inactivation of DNA replication origins by the cell cycle regulator, trigonelline, in root meristems of Lactuca sativa. Planta.

[24] Jacob V., Chernyavskaya Y., Chen X.T., Tan P.S., Kent B., Hoshida Y., Sadler K.C. (2015). DNA hypomethylation induces a DNA replication-associated cell cycle arrest to block hepatic outgrowth in uhrf1 mutant zebrafish embryos. Development.

[25] Berglund T., Wallström A., Nguyen T.V., Laurell C., Ohlsson A.B. (2017). Nicotinamide; antioxidative and DNA hypomethylation effects in plant cells. Plant Physiol. Bioch..

[26] Cho Y., Njiti V.N., Chen X., Lightfoot D.A., Wood A.J. (2003). Trigonelline concentration in field-grown soybean in response to irrigation. Biol. Plant..

[27] Schwartz L.M., Wood A.J., Gibson D.J. (2014). Trigonelline accumulation in leaves of panicum virgatum seedlings. Nat. Prod. Commun..

[28] Li W., Zhang F.X., Wu R., Jia L.J., Li G.S., Guo Y.L., Liu C.M., Wang G.D. (2017). A novel N-methyltransferase in Arabidopsis appears to feed a conserved pathway for nicotinate detoxification among land plants and is associated with lignin biosynthesis. Plant Physiol..

[29] Chen X., Wood A.J. (2004). Purification and characterization of S-adenosyl-L-methionine nicotinic acid-N-methyltransferase from leaves of Glycine max. Biol. Plant..

[30] Ali N.S., Khuwaja A.K. (2011). Chapter 23—Betel nut (*Areca catechu*) usage and its effects on health. Nuts and Seeds in Health and Disease Prevention.

[31] Wu J., Zhang H., Wang S., Yuan L., Grünhofer P., Schreiber L., Wan Y. (2019). Tissue-specific and maturity-dependent distribution of pyridine alkaloids in Areca triandra. J. Plant Res..

[32] Chung C.M., Kuo T.M., Yeh K.T., Lee C.H., Ko Y.C. (2021). Reduction in and preventive effects for oral-cancer risk with antidepressant treatment. J. Pers. Med..

[33] Lichtenthaler H.K., Miehé J.A. (1997). Fluorescence imaging as a diagnostic tool for plant stress. Trends Plant Sci..

[34] Baker N.R. (2008). Chlorophyll fluorescence: A probe of photosynthesis in vivo. Annu. Rev. Plant Biol..

[35] Murchie E.H., Lawson T. (2013). Chlorophyll fluorescence analysis: A guide to good practice and understanding some new applications. J. Exp. Bot..

[36] De Sousa C.A.F., de Paiva D.S., Casari R.A.D.N., de Oliveira N.G., Molinari H.B.C., Kobayashi A.K., Magalhaes P.C., Gomide R.L., Souza M.T. (2017). A procedure for maize genotypes discrimination to drought by chlorophyll fluorescence imaging rapid light curves. Plant Methods.

[37] Yousef A.F., Ali M.M., Rizwan H.M., Tadda S.A., Kalaji H.M., Yang H., Ahmed M.A.A., Wróbel J., Xu Y., Chen F. (2021). Photosynthetic apparatus performance of tomato seedlings grown under various combinations of LED illumination. PLoS ONE.

[38] Wang C.K., Lee W.H., Peng C.H. (1997). Contents of phenolics and alkaloids in *Areca catechu* Linn. during maturation. J. Agr. Food Chem..

[39] Holdsworth D.K., Jones R.A., Self R. (1998). Volatile alkaloids from *Areca catechu*. Phytochemistry.

[40] Peng W., Liu Y.J., Wu N., Sun T., He X.Y., Gao Y.X., Wu C.J. (2015). *Areca catechu* L. (Arecaceae): A review of its traditional uses, botany, phytochemistry, pharmacology and toxicology. J. Ethnopharmacol..

[41] Su Z., Ma X., Guo H.H., Sukiran N.L., Guo B., Assmann S.M., Ma H. (2013). Flower development under drought stress: Morphological and transcriptomic analyses reveal acute responses and long-term acclimation in *Arabidopsis*. Plant Cell.

[42] Anjum S.A., Ashraf U., Tanveer M., Khan I., Hussain S., Shahzad B., Zohaib A., Abbas F., Saleem M.F., Ali I. (2017). Drought induced changes in growth, osmolyte accumulation and antioxidant metabolism of three maize hybrids. Front. Plant Sci..

[43] Szabados L., Savoure A. (2010). Proline: A multifunctional amino acid. Trends Plant Sci..

[44] Zulfiqar F., Akram N.A., Ashraf M. (2019). Osmoprotection in plants under abiotic stresses: New insights into a classical phenomenon. Planta.

[45] Wu J., Cui C., Zhang H., Liu D.J., Schreiber L., Qin W.Q., Wan Y.L. (2021). Identifying new compounds with potential pharmaceutical and physiological activity in *Areca catechu* and *Areca triandra* via a non-targeted metabolomic approach. Phytochem. Anal..

[46] Zhang X.R., Henriques R., Lin S.S., Niu Q.W., Chua N.H. (2006). Agrobacterium-mediated transformation of Arabidopsis thaliana using the floral dip method. Nat. Protoc..

