# Supplementary material for: Overexpression of a Gene Encoding Trigonelline Synthase from Areca catechu L. Promotes Drought Resilience in Transgenic Arabidopsis
Source: Plants (Basel). 2022 Feb 11;11(4):487. doi: 10.3390/plants11040487 (PMC8877179; doi:10.3390/plants11040487)
Supplement: Supplementary file 1 [file plants-11-00487-s001.zip › plants-1564700-Supplementary.pdf]

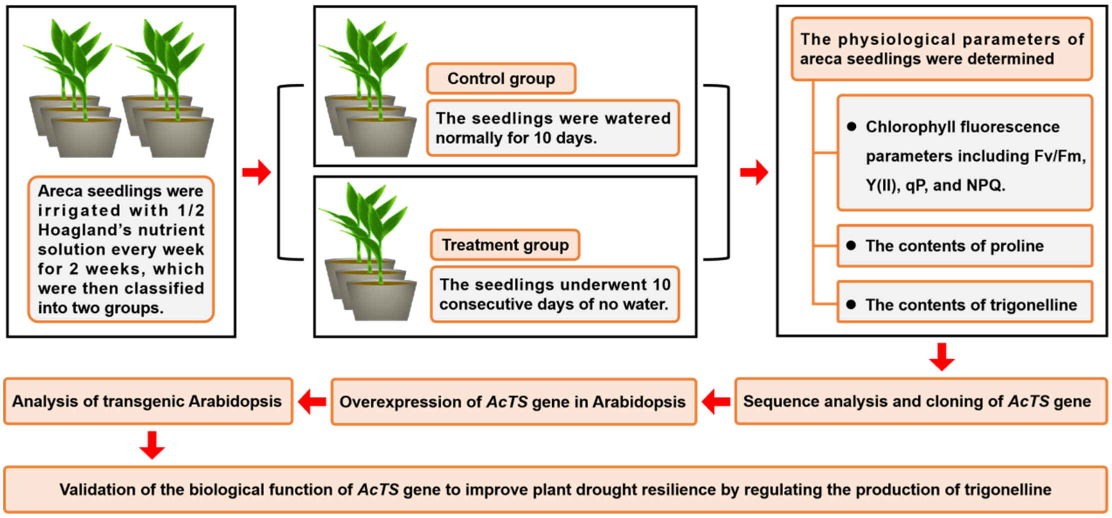


Supplementary Figure S1. The flow chart of this study

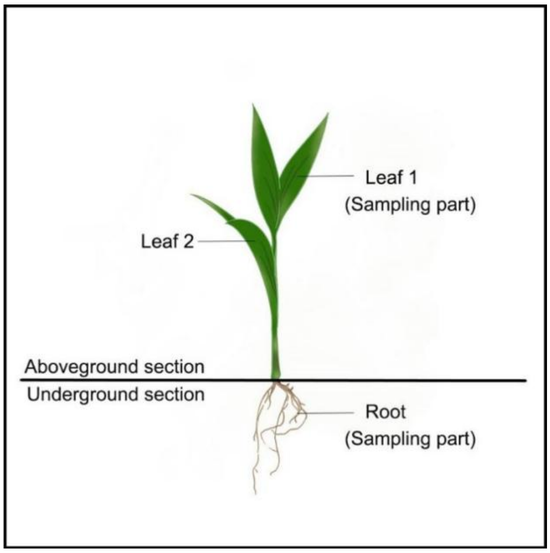


Supplementary Figure S2. Schematic diagram of areca seedling. The roots and the first leaf from the top of each seedling were collected at 0, 2, 4, 6, 8 and 10 days after treatment, which were immediately frozen in liquid nitrogen and stored at -80 °C until use.

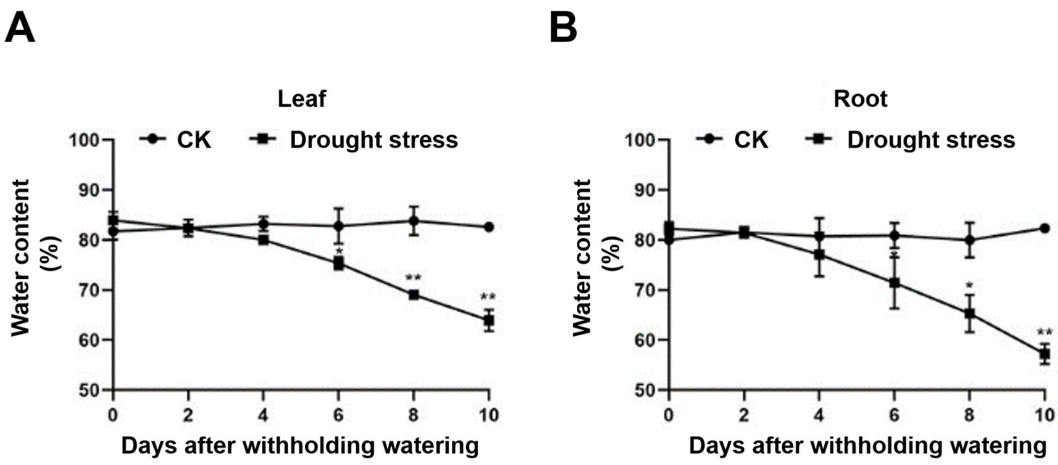


Supplementary Figure S3. The water content in the leaf and root of areca seedling. *: P value < 0.05; **: P < 0.01.

Supplementary Table S1. The primers used for PCR

| Primer name | Primer direction | Primer sequences |
|---|---|---|
| AcTS(KpnI)-1300F | Forward primer | GGGGTACCATGGGGTTGAACG TAGAGCAAGTTTTTCA |
| AcTS(BamHI)-1300R | Reverse primer | CGGGATCCTCACATTTTTGTCT TCAAAGAGATGACAAAAACAG |

Note: The underlined sequences represent restriction enzyme sites, namely KpnI and BamHI.

Supplementary Table S2. The primers used for qRT-PCR

| Gene name | Primer direction | Primer sequences | Product length (bp) |
|---|---|---|---|
| *AcTS* | Forward | TGCTCTTCCGGTCCAAACAC | 228 |
| | Reverse | GCCAGGCACTCCCACAACAT | |
| *Actin* | Forward | GTATCGTGCTTGATTCTGG | 167 |
| | Reverse | GCTACTCTTGGCTGTCTCC | |

Supplementary seq 1. The full length CDS sequences of *AcTS*

ATGGGGTTGAAGGTAGAGCAAGTTTTTCACATGGTTGGAGGGGCTGGAGAGACCAGCTACGCC

ACCAACTCCAGGCTTCAAGAGAAGGCAATATTTGAAGCAAAGCCCATAGTGGAGGAGGCCGTG

AGAGAGGTCTATAAGACTGTCCTGCCTGAGAGCATGGTGGCGGCTGACTTGGGTTGCTCTTCCG

GTCCAAACACATTTCACGTGGTCTTCGAGGTGATCGATATCGTCGACGATCAGAGCCGAAGGCT

GGGACGGCCCCCGCCGGAGATCCAGTTCTTCTTGAACGATCTCCCGGGGAATGACTTCAACAAT

ATTTTTCAGTCTTTGGAAGGATACGAGAAGAAATTAAAGGAAGAGAAGGGAGAGCGGATTCTG

CCGTTCTATGTTGTGGGAGTGCCTGGCTCTTTCTATGGTAGGCTTTTCCCCTCTGGAAGCGTTCAT

TTCTTCCATTCTTCCTACTGTCTCATGTGGCTCTCCCAGGTTCCTGCGGGAGTTGAGAGTAACAC

CGGTGTTCCATTAAATGAGGGAAACATCTACATATGGGAGACAAGTCCACCTAGTGTGGTGAAA

GCATATCAAGAGCAATACCGGAGGGACTTCACCACTTTTCTCGAGTCCCGTTTCCAAGAACTCA

GTTTCGGAGGACGAATGGTCTTAACATTTTTGGGAAGGACGAGCAAAGATGCACGAAGTGGAG

AACTGAGCAAGCTTTGGGGACAACTGGCTGAGGCATTCAATGCTATGGTCCTAGAGGGTATCAT

ACAAAAGGAAAAGGTGGATGCATTCAATGTGCCTTTTTATGCACCTTCCATGGAGGAAGTGAAG

GCAGTGATACAAAGCGTAGGATTATTTGATCTCGATCGAGTACAAATATTCAAGTCAAACTGGG

ATCCACTTGATGACTCTACTGATGATTACGTACATGATAATTTTCTAAGCGGGAACAATGCGGCA

AAGACTATGAGAGCAGTGATGGAACCCGTGATTGAACGTCACTTTGGGGAGCACATACTTGATG

ATTTGTTTTCAAGATATGCAAAAAATGTCGCAAGGCACCTCTTGAAGGAGAAGACCAAGCATCC

TGTTTTTGTCATCTCTTTGAAGACAAAAATGTGA

Supplementary seq 2. Amino acid sequences of AcTS

MGLKVEQVFHMVGGAGETSYATNSRLQEKAIFEAKPIVEEAVREVYKTVLPESMVAADLGCSSGPNTF

HVVFEVIDIVDDQSRRLGRPPPEIQFFLNDLPGNDFNNIFQSLEGYEKKLKEEKGERILPFYVVGVPGSF

YGRLFPSGSVHFFHSSYCLMWLSQVPAGVESNTGVPLNEGNIYIWETSPPSVVKAYQEQYRRDFTTFL

ESRFQELSFGGRMVLTFLGRTSKDARSGELSKLWGQLAEAFNAMVLEGIIQKEKVDAFNVPFYAPSME

EVKAVIQSVGLFDLDRVQIFKSNWDPLDDSTDDYVHDNFLSGNNAAKTMRAVMEPVIERHFGEHIL

DDLFSRYAKNVARHLLKEKTKHPVFVISLKTKM*

Supplementary seq 3. The full length CDS sequences of *CaTS1*

ATGGAGCTCCAAGAAGTCCTGCATATGAATGGAGGCGAAGGCGATGCAAGCTACGCCAAGAAT

TCATCCTTCAATCAACTGGTTCTCGCCAAGGTGAAACCTGTCCTTGAACAATGCGTAGGGGAATT

GTTGCGGGCCAACTTGCCCAACATCAACAAGTGCATTAAAGTTGCGGATTTGGGATGCGCTTCC

GGACCAAACACACTTTTAACAGTTCGGGACATTGTACAAAGTATTGACAAAGTTAGGCAAGAAA

TGAAGAATGAATTAGAACGTCCCACCATTCAGGTTTTTCTGACTGATCTTTTCCAAAATGATTTCA

ATTCGGTTTTCATGTTGCTGCCAAGTTTCTACCGCAAACTTGAGAAAGAAAATGGACGCAAGAT

AGGATCGTGCCTAATAGCCGCAATGCCTGGCTCTTTCCACGGCAGACTCTTCCCCGAGGAGTCA

ATGCATTTTTTACACTCTTCTTACAGTCTTCAATTTTTATCCCAGGTTCCCAGCGGTTTGGTGACTG

AATTGGGGATCACTGCGAACAAAAGGAGCATTTACTCTTCCAAAGCAAGTCCTCCGCCCGTCCA

GAAGGCAAATTTGGATCAATTTACGAAAGATTTTACCACATTTTTAAGGATTCGTTCGGAAGAGT

TGCTTTCACGCGGCCGAATGCTCCTTACTTGCATTTGCAAAGGAGATGAATTCGACGGCCCGAA

TACCATGGACTTACTTGAGATGGCAATAAACGACTTGGTTGTTGAGGGACATCTGGAGGAAGAA

AAATTGGACAGTTTCAATGTTCCAATCTATGCAGCTTCAGTAGAAGAATTAAAGTGCATAGTTGA

GGAGGAAGGTTCTTTTGAAATTTTGTACTTGGAGACTTTTAAGCTCCGTTATGATGCTGGCTTCTC

TATTGATGATGATTGCCAAGTAAGATCCCATTCCCCAGAATACAGCGATGAACATGCTAGAGCA

GCGCATGTGGCATCATTACTTAGATCAGTTTACGAACCCATCCTCGCAAATCATTTTGGAGAAGC

TATTATACCTGACATATTCCACAGGTTTGCGACGAATGCAGCAAAGGTTATCCGCTTGGGCAAA

GGCTTCTATAATAATCTTATCATTTCTCTTGCCAAAAAACCAGAGAAGTCAGACATATAA

Supplementary seq 4. Amino acid sequences of CaTS1

MELQEVLHMNGGEGDASYAKNSSFNQLVLAKVKPVLEQCVGELLRANLPNINKCIKVADLGCASGP

NTLLTVRDIVQSIDKVRQEMKNELERPTIQVFLTDLFQNDFNSVFMLLPSFYRKLEKENGRKIGSCLIAA

MPGSFHGRLFPEESMHFLHSSYSLQFLSQVPSGLVTELGITANKRSIYSSKASPPPVQKANLDQFTKDF

TTFLRIRSEELLSRGRMLLTCICKGDEFDGPNTMDLLEMAINDLVVEGHLEEEKLDSFNVPIYAASVEELK

CIVEEEGSFEILYLETFKLRYDAGFSIDDDCQVRSHSPEYSDEHARAAHVASLLRSVYEPILANHFGEAIIP

DIFHRFATNAAKVIRLGKGFYNNLIISLAKKPEKSDI

Supplementary seq 5. The full length CDS sequences of *CaTS2*

ATGGAGCTCCAAGAAGTCCTGCATATGAATGGAGGCGAAGGCGAAGCAAGCTACGCCAAGAA

TTCATCCTTCAATCAACTGGTTCTCGCCAAGGTGAAACCTGTCCTTGAACAATGCGTACGGGAAT

TGTTGCGGGCCAACTTGCCCAACATCAACAAGTGCATTAAAGTTGCAGATTTGGGATGCGCTTC

CGGACCAAACACACTTTTAACCGTTTGGGACACTGTACAAAGTATTGACAAAGTTAGGCAAGAA

ATGAAGAATGAATTAGAACGTCCCACCATTCAGGTTTTTCTGACTGATCTTTTCCAAAATGATTTC

AATTCGGTTTTCATGCTGCTGCCAAGCTTCTACCGCAAACTTGAGAAAGAAAATGGACGCAAAA

TAGGATCGTGCCTAATAGCCGCAATGCCTGGCTCTTTCCACGGCAGACTCTTCCCCGAGGAGTC

CATGCATTTTTTACACTCTTCTTACAGTCTTCAGTTTTTATCCCAGGTTCCCAGCGGTTTGGTGACT

GAATTGGGGATCACTGCGAACAAAAGGAGCATTTACTCTTCCAAAGCAAGTCCTCCGCCCGTCC

AGAAGGCATATTTGGATCAATTTACGAAAGATTTTACCACATTTTTAAGGATGCGTTCGGAAGAG

TTGCTTTCACGTGGCCGAATGCTCCTTACTTGCATTTGTAAAGGAGATGAATGCGACGGCCCGA

ATACCATGGACTTACTTGAGATGGCAATAAACGACTTGGTTGCTGAGGGACGTCTGGGGGAAG

AAAAATTGGACAGTTTCAATGTTCCAATCTATACAGCTTCAGTAGAAGAAGTAAAGTGCATGGTT

GAGGAGGAAGGTTCTTTTGAAATTTTATACTTGCAGACTTTTAAGCTCCGTTATGATGCTGGCTTC

TCTATTGATGATGATTGCCAAGTAAGATCCCATTCCCCAGTATACAGCGATGAACATGCTAGAG

CAGCGCATGTGGCATCATTAATTAGATCAGTTTACGAACCCATCCTAGCAAGTCATTTTGGAGAA

GCTATTATACCTGACATATTCCACAGGTTTGCGACGAATGCAGCAAAGGTTATCCGCTTGGGCA

AAGGCTTCTATAATAATCTTATCATTTCTCTTGCCAAAAAACCAGAGAAGTCAGACATATAA

## Supplementary seq 6. Amino acid sequences of CaTS2

MELQEVLHMNGGEGEASYAKNSSFNQLVLAKVKPVLEQCVRELLRANLPNINKCIKVADLGCASGP

NTLLTVWDTVQSIDKVRQEMKNELERPTIQVFLTDLFQNDFNSVFMLLPSFYRKLEKENGRKIGSCLIA

AMPGSFHGRLFPEESMHFLHSSYSLQFLSQVPSGLVTELGITANKRSIYSSKASPPPVQKAYLDQFTKD

FTTFLRMRSEELLSRGRMLLTCICKGDECDGPNTMDLLEMAINDLVAEGRLGEEKLDSFNVPIYTASVE

EVKCMVEEEGSFEILYLQTFKLRYDAGFSIDDDCQVRSHSPVYSDEHARAAHVASLIRSVYEPILASHFG

EAIIPDIFHRFATNAAKVIRLGKGFYNNLIISLAKKPEKSDI*

## Supplementary seq 7. The full length CDS sequences of *AtTS*

ATGGAGAACGAAAGCTCAGAGAGTAGAAACAGAGCTCGTCTTGCCATTATGGAGCTTGCTAAC

ATGATTAGCGTTCCCATGTCTCTCAATGCCGCCGTGCGACTAGGCATTGCCGACGCCATTTGGA

ACGGCGGAGCCAATTCTCCTCTCTCTGCCGCCGAGATCCTCCCTCGCCTCCACCTACCATCTCA

CACTACCATTGGTGGCGACCCCGAGAATCTTCAGCGTATACTTCGGATGCTCACCAGCTACGGT

GTCTTCTCCGAACACCTTGTTGGATCCATTGAGAGGAAATACTCTCTTACGGACGTCGGAAAAA

CTCTTGTAACCGACTCCGGCGGCCTCTCTTACGCTGCCTACGTCCTCCAACATCACCAGGAGGC

GTTGATGCGAGCATGGCCACTAGTTCACACGGCAGTGGTGGAGCCGGAGACAGAGCCGTACGT

GAAAGCAAACGGCGAGGCGGCATACGCTCAGTATGGGAAAAGTGAGGAGATGAATGGTCTAA

TGCAAAAGGCAATGTCTGGCGTATCTGTACCGTTCATGAAAGCTATATTAGACGGCTACGATGG

GTTTAAATCAGTGGATATTTTGGTTGACGTAGGAGGTAGTGCAGGGGATTGTCTGCGTATGATCC

TTCAACAATTTCCTAACGTCCGTGAAGGGATTAATTTCGATTTACCTGAAGTTGTTGCCAAAGCC

CCCAATATTCCTGGAGTGACTCACGTGGGTGGGGATATGTTCCAATCAGTTCCTAGCGCTGACG

CTATCTTCATGAAGTGGGTGTTAACGACATGGACGGATGAAGAATGCAAGCAGATAATGAAGA

ATTGCTACAACGCGTTACCAGTTGGAGGAAAGCTGATTGCGTGTGAGCCGGTCTTGCCTAAGGA

AACCGATGAAAGTCACCGGACTCGTGCCTTGTTAGAAGGTGACATCTTTGTCATGACCATCTATA

GAACCAAAGGTAAGCATAGAACCGAAGAAGAGTTTATAGAGCTTGGTCTCTCCGCGGGATTCC

CTACTTTTCGACCTTTCTACATTGATTACTTCTACACCATCTTAGAGTTTCAGAAGTAA

Supplementary seq 8. Amino acid sequences of AtTS

MENESSESRNRARLAIMELANMISVPMSLNAAVRLGIADAIWNGGANSPLSAAEILPRLHLPSHTTIG

GDPENLQRILRMLTSYGVFSEHLVGSIERKYSLTDVGKTLVTDSGGLSYAAYVLQHHQEALMRAWPLV

HTAVVEPETEPYVKANGEAAYAQYGKSEEMNGLMQKAMSGVSVPFMKAILDGYDGFKSVDILVDVG

GSAGDCLRMILQQFPNVREGINFDLPEVVAKAPNIPGVTHVGGDMFQSVPSADAIFMKWVLTTWTD

EECKQIMKNCYNALPVGGKLIACEPVLPKETDESHRTRALLEGDIFVMTIYRTKGKHRTEEEFIELGLSAG

FPTFRPFYIDYFYTILEFQK*

Supplementary seq 9. The full length CDS sequences of *GmTS*

ATGGAGAAAGAGGAGAGCACGGAACAGCGAAAGCAAGCGAGGCTTGCCATTATGGAGCTCGC

CAACATGATAAGCGTTCCCATGGCGCTAAACGCCGTCGTTCGCCTCAACGTCGCCGACGCCATC

TGGCAAGGCGGCGCCAACAACCCCCTCTCCGCCGCTGAGATCCTCCCCCGCCTCCTTCCCGCC

GGCGGCGGCGACGCCGAGAACCTCCAGCGGCTCCTCCGGATGCTGGCCAGTTACGGCGTCTTC

TACGAGCACCTCTCCGCCGGCGAGCGGAAGTACTCCCTCACCGACGTCGGCAAAACGCTTGTC

ACCGATGAACAAGGCCTGTCGTACGCGCATTACGTGCTCCAACACCACCAGGATGCGTTGATG

AGAGCGTGGCCAATGGTGCACGAGGCAGTAGTGGACCCTACAAAGGAGCCCTTCGAGAGGGC

AAATGGGGAGCCAGCATATGGATACTATCTGAAGCACCCAGAGATGAATGACCTAATGGTGAG

GGCCATGTCAGGGGTGTCAGTGCCCTTCATTAGGGCCATGTTGGAGGGCTATGATGGCTTTCAA

GGTGTGGAGAAGCTTGTGGATGTGGGTGGTAGTGGCGGTGATTGCCTTCGTATGATCTTGGAAA

AACACCCCACCATCAAAGAAGGGATCAACTTTGACCTACCTGAAGTTGTGGCCAAAGCCCCAC

AAATCCCATTTGTAACCCATGTGGGTGGTGACATGTTCAAGTTTATTCCCCAAGGAGATGCTATC

TTCATGAAGTGGGTGCTAACAACATGGACTGATGAAGAATGCAAGCACATAATGCAGAACTGTC

ACAAGGCACTCCCTGAGGGAGGAAAACTAATAGCATGTGAGCCAGTGCTCCCGGAGGACTCAG

ACGAGAGTCACAGAACGAGGGCATTGCTTGAAGGTGACATTTTTGTGATGACAATCTACAGAGC

CAAAGGGAAGCACAGGACTGAAGAACAGTTCAGGCAATTAGCCATTGATGCAGGCTTCCCTCG
TTTCAGAGCCTTCCATGTTGACCATTTCTACACTGTCCTTGAGTTTCAGAAATGA

Supplementary seq 10. Amino acid sequences of GmTS

MEKEESTEQRKQARLAIMELANMISVPMALNAVVRLNVADAIWQGGANNPLSAAEILPRLLPAGGG
DAENLQRLLRMLASYGVFYEHLSAGERKYSLTDVGKTLVTDEQGLSYAHYVLQHHQDALMRAWPM
VHEAVVDPTKEPFERANGEPAYGYYLKHPEMNDLMVRAMSGVSVPFIRAMLEGYDGFQGVEKLVDV
GGSGGDCLRMILEKHPTIKEGINFDLPEVVAKAPQIPFVTHVGGDMFKFIPQGDAIFMKWVLTTWTDE
ECKHIMQNCHKALPEGGKLIACEPVLPEDSDESHRTRALLEGDIFVMTIYRAKGKHRTEEQFRQLAIDA
GFPRFRAFHVDHFYTVLEFQK*

Supplementary seq 11. The full length CDS sequences of *OsTS*

ATGGGCGGCGGAGGAGACGGCGAACTGTCCCCGGCGGAGGCCAGGCTGGCGATGATGGAGCT
CGCCAACATGATCTCCGTCCCCATGGCGCTCACCGCCGTCATCCGCCTCGGCGTCCCCGCCAA
GCTCTGGGCGGGAGGCGCCAACGCCCCGCTCGCCGCCGCCGACCTCCTCCCCGCGGGCCACC
CGGACCCCTCCGTCCTCGAGCGCCTCCTCCGCCTCCTCGCCTCCCGCGGCGTCTTCTCCGAGCA
CACCGGATCATCCTCCCCCTCCCCGCGCCGCTTCTCGCTCACCGCCGTCGGCCGCACCCTCGTC
CCCGGCGGCGGCGGTAGCCCCTCCGGCTCCGGCGCGTCCTACGCCGACTACGTTCTGCAGCAC
CACCAGGATGCGCTCGTCCGCGCCTGGCCCCTCCTCCACGAGGCCGTCCTCGACCCCTCCGGC
CCCGAGCCCTTCGCCCGCGCCAACGCCGGCGTCCCCGCCTACGCCTACTACGGCAAGGACAG
GGAGGCGAACGAGGTGATGCTCCGGGCGATGACGGGGGTGTCGGAGCCGTTCATGGAGGCGC
TGCTGGAGGGCTACGGCGACGGCGGGTTCGAGGGGGTGAGCACGCTGGTGGACGTGGGAGGC
AGCTCCGGCGCGTGCCTGGAGATGATCATGCGGCGGGTCCGCACCATCCGGGACGGCGTCAA
CTTCGACCTGCCCGACGTCGTCGCGGCGGCGCCGCCCATTCCCGGAGTGAGGCATGTTGGCGG
GGACATGTTCAAGTCCATCCCCTCCGGTGATGCCATCTTCATGAAGTGGGTTCTGACGACATGG
ACGAACGAGGAGTGCACGGCGATCCTGAGCAACTGCCACAAGGCGCTGCCGGGCGGCGGGAA
GGTGATCGCCTGCGAGCCGGTGGTGCCGGACACGACGGACGGCAGCACGAGGACGAGGGCG
CTGCTGGAGAACGACATCTTCGTCATGGCCACCTACCGGACTCAGGGCAGGGAGCGATCCGAG
GAGGAGTTCCGCCACCTCGGCCTCGCCGCCGGCTTCGCCTCCTTCCGGGCCATCTACCTCGACC
CTTTCTACGCTGTCCTCGAGTACACCAAGTAG

Supplementary seq 12. Amino acid sequences of OsTS

MGGGGDGELSPAEARLAMMELANMISVPMALTAVIRLGVPAKLWAGGANAPLAAADLLPAGHPD

PSVLERLLRLLASRGVFSEHTGSSSPSPRRFSLTAVGRTLVPGGGGSPSGSGASYADYVLQHHQDALV

RAWPLLHEAVLDPSGPEPFARANAGVPAYAYYGKDREANEVMLRAMTGVSEPFMEALLEGYGDGG

FEGVSTLVDVGGSSGACLEMIMRRVRTIRDGVNFDLPDVVAAAPPIPGVRHVGGDMFKSIPSGDAIF

MKWVLTTWTNEECTAILSNCHKALPGGGKVIACEPVVPDTTDGSTRTRALLENDIFVMATYRTQGRE

RSEEEFRHLGLAAGFASFRAIYLDPFYAVLEYTK*
